# Advances in Natural Products and Their Biological Activities

**DOI:** 10.3390/molecules31111849

**Published:** 2026-05-28

**Authors:** Irwin Rose Alencar de Menezes, Henrique Douglas Melo Coutinho, Almir G. Wanderley, Jaime Ribeiro-Filho, Lucindo Quintans-Júnior, Jackson Roberto Guedes da Silva Almeida, Laurent Picot

**Affiliations:** 1 Department of Biological Chemistry, Regional University of Cariri, Crato CEP 63105-000, Brazil; hdmcoutinho@gmail.com; 2 Department of Pharmaceutical Sciences, Universidade Federal de São Paulo (UNIFESP), Diadema CEP 09913-030, Brazil; almir.wanderley@unifesp.br; 3 Oswaldo Cruz Foundation (FIOCRUZ), Fiocruz Ceará, R. São José, S/N—Precabura, Eusébio CEP 61773-270, Brazil; jaime.ribeiro@fiocruz.br; 4 Department of Physiology, Federal University of Sergipe, Aracaju CEP 49100-000, Brazil; lucindo@academico.ufs.br; 5 Department of Pharmacy, Federal University of São Francisco Valley, Petrolina CEP 56304-917, Brazil; jackson.guedes@univasf.edu.br; 6 Littoral Environnement et Sociétés UMRi CNRS 7266 LIENSs, La Rochelle Université, 17042 La Rochelle, France; laurent.picot@univ-lr.fr

The exploration of natural products (NPs) as sources of therapeutic agents represents one of the most enduring and promising frontiers in chemistry of NPs and pharmaceutical sciences. Throughout human history, nature has provided an inexhaustible reservoir of bioactive compounds from ancient herbal remedies to modern blockbuster drugs, demonstrating that the chemical diversity found in plants, bacterial, fungi, and marine organisms continues to offer unparalleled opportunities for drug discovery and development. The present volume brings together a remarkable collection of studies published in Molecules—Special Issue “Advances in Natural Products and Their Biological Activities,” showcasing the breadth, depth, and innovation characterizing contemporary research in this vital field.

As guest editors for this Special Issue, we envisioned a platform that would highlight not only the discovery of novel bioactive compounds but also the sophisticated methodologies and mechanistic insights that define modern NPs research focusing on your activity and applications. The response from the scientific community exceeded our expectations, resulting in a diverse compendium of 99 papers that span the globe in terms of both geographical origin of natural materials and the international collaboration of research groups. From the highlands of Central Asia to the coastal forests of Chile, from the mangrove ecosystems of Brazil to the temperate zones of Europe, this collection reflects the extraordinary biodiversity that continues to inspire the pharmacological discovery.

The studies that unlocking Nature’s Chemical Diversity presented the remarkable chemical complexity inherent in natural sources. Several investigations focus on essential oils (EOs) and their volatile constituents, revealing both chemical variability and functional versatility. The work on *Chromolaena odorata* from Vietnam exemplifies this approach, where detailed GC-MS and chiral GC analyses identified a distinct chemotype characterized by α-pinene, geigerene, and germacrene D, while subsequent bioassays revealed potent larvicidal, repellent, and antimicrobial activities [1]. Similarly, comparative analyses of *Thymus vulgaris* and *Origanum* sp. species elucidated the relationship between chemotype, whether thymol-dominant, carvacrol-dominant, or sesquiterpene-rich, and biological efficacy against phytopathogens, providing valuable insights for agricultural applications [2].

Beyond EOs, this collection explores a vast array of metabolites, including polysaccharides, flavonoids, coumarins, alkaloids, terpenoids, and semisynthetic derivatives. The characterization of polysaccharides from *Artemisia ordosica* and *Adenophora tetraphylla* exemplifies the growing recognition of these macromolecules as important immunomodulatory agents, with mechanisms involving Toll-like receptor signaling and modulation of glucose metabolism [3]. Meanwhile, the discovery of novel azaphilone derivatives from *Penicillium* sp. and koninginins from *Trichoderma* sp. highlights the immense potential of endophytic and endolichenic fungi as sources of structurally unique bioactive molecules [4,5].

A particularly compelling theme emerging from this collection is the validation of Traditional Knowledge, similar to that used in folk medicine, and molecular mechanistic elucidation of plants with established ethnopharmacological use. The investigation of *Heteropterys* species from Mexico, traditionally used for mental disorders, demonstrates that antioxidant, anti-inflammatory, and acetylcholinesterase-inhibitory activities may underlie their antidepressant and anxiolytic effects [6]. Similarly, the study of *Baccharis concava*, a medicinal shrub from the Chilean coast, provides phytochemical characterization and antimicrobial data that support its traditional use in wound healing [7]. The systematic review by Barbosa and colleagues on *Plectranthus* species included in this Special Issue exemplifies the power of this integrative approach. By synthesizing data from 45 ethnobotanical and pharmacological studies, the authors identified twelve *Plectranthus* species traditionally used across Africa, South America, and Asia to treat inflammatory conditions, however, only four have been subjected to experimental investigation, highlighting a critical gap between ethnobotanical knowledge and scientific validation [8].

Several contributions delve deeply into the molecular mechanisms, revealing the sophisticated pathways through which natural products exert their effects. The work on physalin F as a calcineurin inhibitor with synergistic immunosuppressive activity when combined with dexamethasone opens new possibilities for combination therapies in autoimmune and allergic conditions [9]. The investigation of 3,3′-dihydroxy-4,5-dimethoxybibenzyl demonstrates the involvement of p38 MAP kinase pathway in cancer cell death, while atractylenolide II was shown to suppress glycolysis and induce apoptosis in endometrial cancer cells through PADI3-ERK signaling blockade [10]. These mechanistic studies not only validate traditional uses but also identify potential molecular targets for future drug development.

The therapeutic scope covered in this volume is remarkably broad, addressing many of the most pressing health challenges of our time. Antimicrobial resistance of microorganism features prominently, with numerous studies evaluating natural products against multidrug-resistant bacterial and fungal pathogens. The development of an essential oil incorporating clove and oregano demonstrates practical translational potential, showing efficacy comparable to synthetic antiseptics against resistant microorganisms. The anti-biofilm activity of *Origanum vulgare* extracts obtained by supercritical fluid extraction further expands the potential applications of natural products in combating persistent infections [11]. The study on *Spondias tuberosa* by Santos and colleagues exemplifies the value of integrating ethnopharmacological knowledge with modern analytical and microbiological techniques to address fungal infections, a growing concern in the context of antifungal resistance, with a dual mechanism of direct antifungal synergy combined with virulence attenuation, which offers a compelling strategy for overcoming antifungal resistance [12].

Cancer therapeutics constitute another major focus, with contributions spanning diverse mechanisms and cancer types. From the cytotoxic activity of naphthoquinone derivatives against breast cancer cells in three-dimensional culture models to the anti-colorectal cancer effects of echinulin derivatives from endolichenic fungi, these studies illustrate the ongoing search for novel anticancer agents with improved efficacy and safety profiles [13]. The comprehensive review of cajaninstilbene acid and its derivatives, as well as the systematic analysis of sinomenine’s bioactivities, provide valuable syntheses of existing knowledge while identifying avenues for future research [14]. These studies highlight the need for clinical research to translate this knowledge into clinical practice.

Methodological innovation represents another strength of this collection. The application of supercritical carbon dioxide extraction for the isolation of coumarins from Pterocaulon polystachyum demonstrates the potential of green technologies to obtain bioactive extracts from plant materials that might otherwise be discarded, thereby embodying circular bioeconomy principles [15]. The investigation of extraction systems and seasonal variation on the pharmacological potential of *Eugenia punicifolia* highlights the critical importance of optimizing extraction parameters to maximize bioactivity, with quantitative NMR spectroscopy (qNMR) providing robust chemical characterization [16].

The development of novel formulations, including the microemulsion of *Chromolaena odorata* essential oil with enhanced larvicidal activity, illustrates how formulation science can improve the practical applicability of natural products [1]. Similarly, the study of salidroside emulsion gels for wound repair represents an important step toward clinical translation [17].

The 99 papers collected in this Special Issue represent a snapshot of the vibrant and diverse field of NPs research at a particular moment in time, yet they also point toward enduring themes that will continue to shape the discipline. The integration of traditional knowledge with modern analytical and biological techniques, the exploration of understudied ecosystems and organisms, the elucidation of molecular mechanisms, and the development of sustainable extraction and formulation strategies throughout this collection.
